# Prevalence and Sociodemographic Covariates of Cervical Cancer and Its Association With Menstrual Irregularities: Findings From the 2017 National Inpatient Sample Database

**DOI:** 10.7759/cureus.18855

**Published:** 2021-10-18

**Authors:** Helen Varghese, Mohammed Abdul Salam Qureshi, Ashwini Ronghe, Ankit Vyas, Romil Singh, Karthik Chamarti

**Affiliations:** 1 Internal Medicine, Ross University School of Medicine, Bridgetown, BRB; 2 Pathology, State University of New York Broome, Binghamton, USA; 3 Internal Medicine, University at Buffalo, Buffalo, USA; 4 Cardiology, Inova Heart and Vascular Institute, Falls Church, USA; 5 Critical Care, Mayo Clinic, Rochester, USA; 6 Internal Medicine, Texas Tech University Health Sciences Center, Odessa, USA

**Keywords:** epidemiology, demographics, hospitalizations, menstrual irregularities, cervical cancer

## Abstract

Background and objective

Menstrual irregularities and sociodemographic factors such as increasing age, Hispanic race, low socioeconomic strata, and low income status are known risk factors for cervical cancer. This study aimed to examine the prevalence of cervical cancer and its association with menstrual irregularities and other known risk factors based on a large nationwide inpatient sample database.

Methods

We used the Nationwide Inpatient Sample (NIS) database for the year 2017 and identified cases where cervical cancer and menstrual irregularities are the primary and co-occurring diagnoses using the International Classification of Diseases, Tenth Revision (ICD-10) codes. Pearson's chi-square test, independent-sample t-test, and multiple logistic regression were used to generate the analysis.

Results

A total of 15,800 (0.19%) female weighted admissions between the age group of 18-55 years reported a diagnosis of cervical cancer. Patients with a diagnosis of menstrual irregularity had a statistically significant higher odds of association [odds ratio (OR): 1.582] for being diagnosed with cervical cancer. The odds of association were also high for the Hispanic race [OR: 1.280, 95% confidence interval (CI): 1.128-1.453]. The odds of a diagnosis of cervical cancer increased with age, with the highest odds being reported for the age group of 46-55 years (95% CI: 12.107-21.171) and the population with lower median household income, with the highest odds being observed for the lowest interquartile range (95% CI: 1.418-1.892).

Conclusion

Based on our findings, a diagnosis of menstrual irregularity, the Hispanic race, increasing age, and lower household income are factors that significantly increased the odds of being diagnosed with cervical cancer.

## Introduction

In the United States, approximately 14,500 new cases of invasive cervical cancer and 4,300 cancer-related deaths occur each year [1]. Cervical cancer ranks as the third most common gynecologic cancer diagnosed in the United States [1]. As per a worldwide analysis pooling in data from 185 countries using the Global Cancer Observatory 2018 database, the total cases of cervical cancer and deaths were approximately 570,000 and 311,000 respectively [2]. The global age-standardized incidence of cervical cancer as per the same study was 13.1 per 100,000 women [2].

The bulk of the research has recently focused on understanding the human papillomavirus (HPV)-related risk factors. As per a study, adenocarcinoma and squamous cell carcinoma share the same risk factors with the pre-invasive disease that corresponds with these two histologies [3]. Non-HPV-related studies have not received the same level of attention. Non-HPV-related risk factors include low socioeconomic status [4], oral contraceptive use [5], cigarette smoking [6], genetics [7], and circumcision trends [8]. We conducted a national study using the Nationwide Inpatient Sample (NIS) data to evaluate the various sociodemographic factors such as age, race, income, and clinical diagnoses attributed to menstrual irregularities, and examine their association with the admission diagnosis of cervical cancer.

## Materials and methods

Data source

We used the Healthcare Cost and Utilization Project (HCUP)'s NIS data for the year 2017 to conduct a retrospective study [9]. It is the largest inpatient database, which covers 4,411 hospitals from 45 states in the United States [9]. State and hospital identifiers were de-identified to protect the privacy of individual patients, physicians, and hospitals [9]. Since the study was conducted based on publicly available de-identified data, an institutional review board approval was not warranted.

Inclusion criteria

We included adult patients (age: >18 years and ≤55 years) of female gender with cervical cancer as one of the admitting diagnosis based on the International Classification of Diseases, Tenth Revision (ICD-10) diagnosis codes ('C530','C531','C538','C539','D060','D061','D067','D069') (Appendix A) [9].

Variables of interest

The sociodemographic variables included in this study were age, sex, race, and median household income for patients' ZIP Code (based on current year) [10]. The following ICD-10 diagnosis codes for menstrual disorders were used to classify a subset of sample based on their menstrual history: 'N897', 'N910', 'N911', 'N912', 'N913', 'N914', 'N915', 'N920', 'N921', 'N922', 'N923', 'N925', 'N926', 'N944', 'N945', 'N946' (Appendix B).

Statistical analysis

Descriptive statistics were used to characterize participant details; chi-square and Fisher's exact tests were used to assess differences in proportions between dichotomous outcome and explanatory variables. Continuous data were analyzed with the help of an independent sample t-test, which allowed us to study the difference in demographics. We used the PROC SURVEYFREQ and PROC SURVEYLOGISTIC tools in the SAS 9.4 software (SAS Institute, Cary, NC) to generate the analyses. These tools allowed us to factor in the weight, cluster, and strata variables as outlined in the NIS dataset [10], allowing us to obtain a national representation of the sample population. Differences in comorbidities were quantified using chi-square tests. We used a binomial logistic regression model to evaluate the odds ratio (OR) and 95% confidence interval (CI). The regression model was adjusted for age, race, and median household income. A p-value <0.05 was used to determine the statistical significance of the test results. All statistical analyses were performed using the SAS 9.4 software.

## Results

We analyzed a total of 1,636,156 observations across 206 strata and 4,444 clusters. A total of 15,800 female weighted admissions in the age group of 18-55 years reported a diagnosis of cervical cancer, which amounted to 0.19% of the total sample. As shown in Table 1, 480 (3.13%) patients with a diagnosis of cervical cancer reported a diagnosis of menstrual irregularities. The majority of the admitted females belonged to the Hispanic race, followed by White and Black. The majority were in the age category of 36-55 years. The prevalence of cervical cancer was higher among patients from the lower household income quartile.

**Table 1:** Characteristics of cervical cancer patients by demographics and menstrual irregularities

Variables	With cervical cancer	Cervical cancer with menstrual irregularities	Cervical cancer without menstrual irregularities	P-value
	Weighted frequency (%)			
Total number of patients	15,800 (0.19%)	480 (3.13%)	15,320 (96.87%)	<0.05
Characteristics				
Sex				
Female	15,800 (0.19%)	480 (3.13%)	15,320 (96.87%)	<0.05
Race				
White	8,415 (0.19%)	280 (0.07%)	8,135 (0.19%)	<0.05
Black	2,835 (0.19%)	80 (0.005%)	2,755 (0.18%)	
Hispanic	2,850 (0.28%)	90 (0.007%)	2,760 (0.21%)	
Asian or Pacific Islander	490 (0.15%)	5 (0.001%)	485 (0.15%)	
Native American	85 (0.13%)	5 (0.00004%)	80 (0.13%)	
Other	695 (0.22%)	10 (0.003%)	685 (0.22%)	
Age at admission, years				
18-25	345 (0.02%)	10 (0.0007%)	335 (0.02%)	<0.05
26-35	3,510 (0.12%)	100 (0.003%)	3,410 (0.12%)	
36-45	5,625 (0.33%)	200 (0.01%)	5,425 (0.32%)	
46-55	5,565 (0.34%)	145 (0.009%)	5,420 (0.33%)	
Median household income, percentile				
0-25th	5,980 (0.23%)	175 (0.007%)	5,805 (0.23%)	<0.05
26th-50th	4,000 (0.19%)	130 (0.006%)	3,870 (0.18%)	
51st-75th	3,265 (0.17%)	85 (0.005%)	3,180 (0.17%)	
76th-100th	2,350 (0.15%)	90 (0.006%)	2,260 (0.15%)	

Odds of association were calculated using the PROC SURVEYLOGISTIC regression method in SAS 9.4 software. The multivariate logistic regression model was controlled for demographic confounders.

As shown in Table 2, patients with a diagnosis of menstrual irregularity had a statistically significant higher odds of association (1.582) for being diagnosed with cervical cancer (Appendix B) (95% CI: 1.264-1.979). With the White race as a reference group, odds of association were statistically significant only for the Hispanic race who had a 1.280 times higher risk of being diagnosed with cervical cancer (95% CI: 1.128-1.453). The odds of a diagnosis of cervical cancer increased with increasing age, with the highest odds being reported for the age group of 46-55 years (OR: 16.01; 95% CI: 12.107-21.171). Odds of association were also higher for the population with lower median household income, with the highest odds being observed for the lowest interquartile range with the highest interquartile range as the reference (OR: 1.638; 95% CI: 1.418-1.892).

**Table 2:** Odds of association for cervical cancer with menstrual irregularities and sociodemographic covariates

Variables	Odds ratio (adjusted)	95% confidence interval		P-value
		Lower	Upper	
Menstrual irregularities				
Absent	Reference			
Present	1.582	1.264	1.979	<0.001
Race (uniform)				
White	Reference			0.002
Black	0.916	0.81	1.036	
Hispanic	1.28	1.128	1.453	
Asian or Pacific Islander	0.933	0.751	1.159	
Native American	0.675	0.379	1.204	
Other	1.321	0.727	2.399	
Age category at the time of admission, years				
18-25	Reference			<0.001
26-35	5.894	4.392	7.91	
36-45	15.83	11.958	20.958	
46-55	16.01	12.107	21.171	
Median household income, percentile				
0-25th	1.638	1.418	1.892	
26th-50th	1.332	1.159	1.532	
51th-75th	1.193	1.033	1.378	
76th-100th	Reference			<0.001

## Discussion

The findings from our study reveal that patients with a diagnosis of menstrual irregularity have a higher odds of being diagnosed with cervical cancer. Higher odds were also observed for the Hispanic race, older age groups, and patients from lower median income households. These figures help us understand the epidemiology of the disease better and will help guide the screening programs and vaccination drives.

Our findings are in line with prior studies. One case-control study conducted among Thai women observed that women with a history of irregular menstruation both in amount and frequency were twice as prone to cervical cancer as normal women.

We noted that the odds of being diagnosed with cervical cancer increased with age. Women in the age group of 36-55 years had the highest odds, which were statistically significant. These findings are in line with results from prior studies, which observed that the mean age at diagnosis of cervical cancer in the United States from 2014 to 2016 was 50 years. From 2014 to 2016, the United States age-adjusted incidence of cervical cancer in girls under the age of 20 years was 0.1 per 100,000, rising to 1.3 per 100,000 in females aged 20-34 years, and peaking at between 2.2 and 2.3 per 100,000 in females aged 35-54 years [11].

With the White race as the reference group, significant odds of association were observed only for the Hispanic group, which reported 1.28 times higher odds of being diagnosed with cervical cancer. No significant association was observed for the Black, Asian or Pacific Islander, Native American, or other racial groups. These findings are in contrast with a United States national survey, which observed a higher magnitude of difference in cervical cancer mortality between Black and White females [1]. This could be due to a discrepancy in the inclusion/exclusion criteria of the survey, which made no differentiation between total and supracervical hysterectomy. The NIS database made no such discrimination as part of its survey design.

The odds of being diagnosed with cervical cancer increased with a decrease in the median household income, with the highest odds of 1.638 (95% CI: 1.418-1.892) being observed for the lowest interquartile range. As per another study, due to rising investments in universal access to screening and treatment, cervical cancer and its associated mortality are on a decline in high-income settings [12].

A significant strength of our study is the utilization of the NIS database. It is the largest nationwide sample of inpatient hospitalization. The large sample size ensured that the power of the study was high and allowed us to detect the differences existing among the patient subgroups with confidence. Since we used the demarcated weights, stratum, and cluster variables, the findings from our analysis are robust enough for extrapolation and generalizability to the much larger general population. To our knowledge, this is one of the first studies to analyze the sociodemographic associations of cervical cancer based on such a large cross-sectional database.

While interpreting the findings of the study, one must take into account its limitations. One should acknowledge that the subject of analysis was entirely composed of patients having an inpatient hospitalization. Re-hospitalization was not accounted for given the nature of the database, which could add to the total disease load. Important characteristics such as patients' complications and comorbidities were not considered. The cross-sectional nature of the database limits us from commenting on the causal inferences. Future studies might benefit from the utilization of longitudinal-based designs and clinical data. Nevertheless, our findings can help guide the existing screening programs and vaccination drives.

## Conclusions

We found that the odds of being diagnosed with cervical cancer were significantly associated with a diagnosis of menstrual irregularity, the Hispanic race, increasing age, and lower household income. Findings from our study are in line with the existing literature, and the specific quantification of the risk factors will further help guide the existing screening programs and vaccination drives.

## Appendices

Appendix A: ICD-10 code description for cervical cancer

C530: Malignant neoplasm of endocervix

C531: Malignant neoplasm of exocervix

C538: Malignant neoplasm of overlapping sites of cervix uteri

C539: Malignant neoplasm of cervix uteri, unspecified

D060: Carcinoma in situ of endocervix

D061: Carcinoma in situ of exocervix

D067: Carcinoma in situ of other parts of cervix

D069: Carcinoma in situ of cervix, unspecified

**Appendix B: ICD-10 code description for menstrual disorders**

N897: Hematocolpos

N910: Primary amenorrhea

N911: Secondary amenorrhea

N912: Amenorrhea, unspecified

N913: Primary oligomenorrhea

N914: Secondary oligomenorrhea

N915: Oligomenorrhea, unspecified

N920: Excessive and frequent menstruation with a regular cycle

N921: Excessive and frequent menstruation with an irregular cycle

N922: Excessive menstruation at puberty

N923: Ovulation bleeding

N925: Other specified irregular menstruation

N926: Irregular menstruation, unspecified

N944: Primary dysmenorrhea

N945: Secondary dysmenorrhea

N946: Dysmenorrhea, unspecified
